# New Psychological Perspectives on Death and Dying—Between Normality and the COVID-19 Emergency

**DOI:** 10.3390/bs12110414

**Published:** 2022-10-27

**Authors:** Ines Testoni

**Affiliations:** 1 Department of Philosophy, Sociology, Education and Applied Psychology (FISPPA), University of Padova, 35122 Padova, Italy; ines.testoni@unipd.it; 2 Emili Sagol Creative Arts Therapies Research Center, University of Haifa, Haifa 3498838, Israel

## 1. On Death and Dying Removal, Death Education and Self-Determination

In recent decades, there has been a constantly increasing preoccupation with physical perfection and the scientific urge to improve life expectancy. However, the inability to cope with death has grown proportionally with the ability to raise levels of health, well-being and lifespan. In a culture where people can live and remain youthful for an increasingly longer time, death is considered a failure, and the weakening of the body due to age or illness has been interpreted as a preventable and avoidable evil [1]. In this time of technological control over the main aspects of human life, there is, probably more than in the past, a need to address all issues inherent to the relationships between life and death. In the academic and clinical worlds, some very significant theoretical and applied research fields are becoming increasingly important. Among them, death education is necessary for the training of health care personnel and also for the community of patients and family members they serve. In an age where death is systematically removed and denied in the language of everyday social life, it is inevitable that people expect medicine to continuously guarantee health. However, people will continue to become ill and medicine is not omnipotent, so it is necessary for both health care personnel and the general population to understand bodily limitations [2].

Research in this area is beginning to secure increasingly significant results, and the first section of this Special Issue is devoted to this very topic. The first article, “’Imagine You Have ALS’: Death Education to Prepare for Advance Treatment Directives” [3], presents the results of qualitative research carried out within a death education project dedicated to advance treatment directives. In this study, participants were asked to empathize with people who had received a diagnosis of amyotrophic lateral sclerosis. The second article, “A Challenge for Palliative Psychology: Freedom of Choice at the End of Life among the Attitudes of Physicians and Nurses” [4], considers a particular aspect of death education, that is, attitudes concerning advance health care directives among Italian physicians and nurses. The third article, “Enhancing Existential Thinking through Death Education: A Qualitative Study among High School Students” [5], discusses overcoming the removal of death-related issues among adolescents and how this experience did not cause anguish nor depression. As previously discussed in the literature [6], the fourth article, “Lack of Truth-Telling in Palliative Care and Its Effects among Nurses and Nursing Students” [7], considers the necessity of death education in university courses to promote future health care workers’ truth-telling when communicating with severely ill patients and their relatives.

One key aspect of death education concerns self-determination and dignity. Awareness of the existential limit makes it possible to value the conditions of illness and restriction of freedoms. To construct dignity in the face of bodily limitations and death, many professionals working in the fields of palliative care, psychiatric illness and restraint engage with *dignity therapy*. The second section of this Special Issue is devoted to this area of innovation and its operationalization with people who are facing death and severe physical limitations. The first article, “The Sense of Dignity at the End of Life: Reflections on Lifetime Values through the Family Photo Album” [8], presents how to improve dignity therapy through the use of family photo albums. The second article, “The Value of Dignity in Prison: A Qualitative Study with Life Convicts” [9], considers the application of dignity therapy in prisons with people who await death due to lack of freedom during a life sentence. The third contribution, “Improving Dignity of Care in Community-Dwelling Elderly Patients with Cognitive Decline and Their Caregivers. The Role of Dignity Therapy” [10], promotes using dignity therapy to treat elderly people who are nearing the ends of their lives.

## 2. On Loss, Bereavement and the COVID-19 Pandemic Crisis

Clinical research in recent years has reflected increasing interest in the topic of loss, grief and bereavement, focusing particularly on resilience and coping factors. The article “The Integration of Stressful Life Experiences Scale and the Inventory of Complicated Spiritual Grief: The Italian Validation of Two Instruments for Meaning-Focused Assessments of Bereavement” [11] discusses an original aspect of loss and grief: the religious crisis, as caused by trauma or the death of a loved one. Another innovative area of study is that of coping with grief through the internet. The article “Grief Iconography between Italians and Americans: A Comparative Study on How Mourning Is Visually Expressed on Social Media” [12] discusses the recent means to express grief through social media posts. The third article, “The Endless Grief in Waiting: A Qualitative Study of the Relationship between Ambiguous Loss and Anticipatory Mourning amongst the Relatives of Missing Persons in Italy” [13], presents a further area of reflection on death and grief. Ambiguous loss is not thoroughly studied, though this article may offer support for this complex form of mourning. Another particular expression of loss and grief is presented in the article “Loss of Close Relationships and Loss of Religious Belonging as Cumulative Ostracism: From Social Death to Social Resurrection” [14]. Ostracism is a psychosocial construct that may explain the experience of loss and mourning suffered by people ostracized from their religious practices.

A particular aspect of bereavement is trauma, and recent experiences related to the COVID-19 pandemic have created a considerable number of people affected by this type of loss. The article “Meaning-Making Coping Methods among Bereaved Parents: A Pilot Survey Study in Sweden” [15] introduces the theme of trauma related to deaths caused by COVID-19. Likewise, the article “A Scoping Review of Interventions for Family Bereavement Care during the COVID-19 Pandemic” [16] summarizes findings from the existing literature regarding bereavement support interventions (i.e., psychosocial and psychotherapeutic interventions) for family caregivers of people who died of COVID-19.

Indeed, at a time when it seems that science and medicine can guarantee an ever-improving state of well-being and ever-decreasing mortality rates, the pandemic forced us to confront our unpreparedness to face death. COVID-19 made the topic of death the first and most salient experience and most significant social issue. A *Lancet* study group assessed the number of deaths due to COVID-19, discovering that the excess death estimate was 18.2 million between 2020 and 2021 [17]. This yields an average global all-age excess mortality rate of 120.3 deaths per 100,000 people, with that of 21 countries exceeding 300 deaths per 100,000 people [17]. Given the significance of this tragic worldwide experience, it was necessary to consider how death overwhelmed the common lives of people in this Special Issue, who now face a massive increased risk of death; how the infodemic disoriented and kept people on constant alert; how the enforcement of social restriction measures to contain the pandemic affected the global population and contributed to the deterioration of mental health and well-being; how communities and health services were overwhelmed; how families reacted to seeing their loved ones leave in an ambulance and never return; and how feelings of loneliness and social isolation, as well as the rarefaction of social relations, caused severe suffering due to death anxiety.

The final section of this Special Issue is devoted to these themes, with particular attention given to coping and resilience strategies. The first article, “‘You’re Not Alone for China’: The First Song in Times of COVID-19 to Keep the Faith in a World Crying in Silence” [18], discusses how music videos were a spontaneous artistic expression which permitted support during the traumatic pandemic events and the mourning process in China. “Crafting Life Stories in Photocollage: An Online Creative Art-Based Intervention for Older Adults” [19] considers how creative art therapy experiences provided a safe and creative environment for older adults to process life experiences and maintain personal growth while aging during the pandemic. The article “From Fear to Hopelessness: The Buffering Effect of Patient-Centered Communication in a Sample of Oncological Patients during COVID-19” [20] describes the severe distress oncological patients endured because of the fear of death caused by the pandemic. The fourth article, “Psychopathological Impact and Resilient Scenarios in Inpatients with Schizophrenia Spectrum Disorders Related to Covid Physical Distancing Policies: A Systematic Review” [21], considers another critical group of fragile patients. This contribution describes how the COVID-19 pandemic posed great challenges to the healthcare community and how the lack of social contact, as well as the disruptions to daily life, exacerbated anxiety and depressive symptoms in these patients. Finally, the last article, “Journalistic Denial of Death during the Very First Traumatic Period of the Italian COVID-19 Pandemic” [22], examines how denial and fear of death influenced the media in the first phase of the pandemic in Italy.

This Special Issue reviews these varied and evolving research challenges, covering many aspects of death and dying and presenting the most innovative contributions to the literature. For this reason, it represents a crucial volume for health professionals and scholars who work in the field of death and dying.

## References

[1] Testoni I., Lazzarotto Simioni J., Di Lucia Sposito D. (2013). Representation of death and social management of the limit of life: Between resilience and irrationalism. Nutr. Ther. Metab..

[2] Rodríguez Herrero P., De la Herrán Gascón A., Pérez-Bonet G., Sánchez-Huete J.C. (2020). What do teachers think of death education?. Death Stud..

[3] Testoni I., Palazzo L., Calamarà N., Rossi G., Wieser M.A. (2021). “Imagine You Have ALS”: Death Education to Prepare for Advance Treatment Directives. Behav. Sci..

[4] Testoni I., Bortolotti C., Pompele S., Ronconi L., Baracco G., Orkibi H. (2020). A Challenge for Palliative Psychology: Freedom of Choice at the End of Life among the Attitudes of Physicians and Nurses. Behav. Sci..

[5] Testoni I., Palazzo L., De Vincenzo C., Wieser M.A. (2020). Enhancing Existential Thinking through Death Education: A Qualitative Study among High School Students. Behav. Sci..

[6] Zamperini A., Paoloni C., Testoni I. (2015). The emotional labor of nursing: Critical incidents and coping strategies. Assist. Inferm. E Ric..

[7] Testoni I., Wieser M.A., Kapelis D., Pompele S., Bonaventura M., Crupi R. (2020). Lack of Truth-Telling in Palliative Care and Its Effects among Nurses and Nursing Students. Behav. Sci..

[8] Testoni I., Baroni V., Iacona E., Zamperini A., Keisari S., Ronconi L., Grassi L. (2020). The Sense of Dignity at the End of Life: Reflections on Lifetime Values through the Family Photo Album. Behav. Sci..

[9] Testoni I., Marrella F., Biancalani G., Cottone P., Alemanno F., Mamo D., Grassi L. (2020). The Value of Dignity in Prison: A Qualitative Study with Life Convicts. Behav. Sci..

[10] Ounalli H., Mamo D., Testoni I., Belvederi Murri M., Caruso R., Grassi L. (2020). Improving Dignity of Care in Community-Dwelling Elderly Patients with Cognitive Decline and Their Caregivers. The Role of Dignity Therapy. Behav. Sci..

[11] Neimeyer R.A., Testoni I., Ronconi L., Biancalani G., Antonellini M., Dal Corso L. (2021). The Integration of Stressful Life Experiences Scale and the Inventory of Complicated Spiritual Grief: The Italian Validation of Two Instruments for Meaning-Focused Assessments of Bereavement. Behav. Sci..

[12] Cupit I.N., Sapelli P., Testoni I. (2021). Grief Iconography between Italians and Americans: A Comparative Study on How Mourning Is Visually Expressed on Social Media. Behav. Sci..

[13] Testoni I., Franco C., Palazzo L., Iacona E., Zamperini A., Wieser M.A. (2020). The Endless Grief in Waiting: A Qualitative Study of the Relationship between Ambiguous Loss and Anticipatory Mourning amongst the Relatives of Missing Persons in Italy. Behav. Sci..

[14] Zamperini A., Menegatto M., Mostacchi M., Barbagallo S., Testoni I. (2020). Loss of Close Relationships and Loss of Religious Belonging as Cumulative Ostracism: From Social Death to Social Resurrection. Behav. Sci..

[15] Ahmadi F., Zandi S. (2021). Meaning-Making Coping Methods among Bereaved Parents: A Pilot Survey Study in Sweden. Behav. Sci..

[16] Laranjeira C., Moura D., Salci M.A., Carreira L., Covre E., Jaques A., Cuman R.N., Marcon S., Querido A. (2022). A Scoping Review of Interventions for Family Bereavement Care during the COVID-19 Pandemic. Behav. Sci..

[17] Wan H., Paulson K.R., Pease S.A., Watson S., Murray C. (2022). Estimating excess mortality due to the COVID-19 pandemic: A systematic analysis of COVID-19-related mortality, 2020–2021. Lancet.

[18] Giménez-Llort L. (2022). ‘You’re Not Alone for China’: The First Song in Times of COVID-19 to Keep the Faith in a World Crying in Silence. Behav. Sci..

[19] Keisari S., Piol S., Elkarif T., Mola G., Testoni I. (2022). Crafting Life Stories in Photocollage: An Online Creative Art-Based Intervention for Older Adults. Behav. Sci..

[20] Rossi A.A., Marconi M., Taccini F., Verusio C., Mannarini S. (2021). From Fear to Hopelessness: The Buffering Effect of Patient-Centered Communication in a Sample of Oncological Patients during COVID-19. Behav. Sci..

[21] Caponnetto P., Benenati A., Maglia M.G. (2021). Psychopathological Impact and Resilient Scenarios in Inpatient with Schizophrenia Spectrum Disorders Related to Covid Physical Distancing Policies: A Systematic Review. Behav. Sci..

[22] Solomon S., Rostellato D., Testoni I., Calabrese F., Biasco G. (2021). Journalistic Denial of Death during the Very First Traumatic Period of the Italian COVID-19 Pandemic. Behav. Sci..

